# YCT-529: a promising oral non-hormonal male contraceptive and its implications for family planning

**DOI:** 10.1097/MS9.0000000000004801

**Published:** 2026-02-18

**Authors:** Hamza Sajid, Fatima Imran, Noor Un Nisa Irshad, Sakan Binte Imran

**Affiliations:** a Department of Internal Medicine, Allama Iqbal Medical College, Lahore, Pakistan; b Department of Internal Medicine, Jinnah Sindh Medical University, Karachi, Pakistan; c Department of Internal Medicine, Sir Salimullah Medical College, Dhaka, Bangladesh

*To the Editor,*

YCT-529, a selective retinoic acid receptor-α (RAR-α) antagonist, has recently demonstrated strong and reversible suppression of spermatogenesis and has begun first in human safety testing. It provides a way to use a non-hormonal method of male contraception with negligible systemic endocrine effects by selectively targeting vitamin-A-dependent sperm maturation pathways. This intervention may finally close the long-standing gender gap in responsibility for contraception^[1,2]^.

The majority of contraceptive options are still geared toward women. Male contraceptive methods like condoms or vasectomy may have significant acceptability, but they also come with reversibility issues. There is still an unmet need for more safe and reversible family planning techniques. Increasing the options for male contraceptives would address societal stratification and clinical gaps in reproductive responsibilities^[3]^.

Unlike hormonal male contraceptive methods, which harm mood, YCT-529 antagonizes RAR-α in the testis, disrupting spermatogonial differentiation and sperm maturation without affecting androgen levels. This spermatogenic suppression and reversibility were noted in primates, and preclinical animal research reports near-complete contraception in mating studies (≈99% efficacy in mice) with full recovery of fertility after treatment cessation. Human contraceptive efficacy has not yet been established, and phase Ib/II studies will evaluate efficacy and reversibility in men, while early human phase 1 safety/pharmacokinetic data show favorable tolerability. YCT-529 may be the first effective oral, non-hormonal male contraceptive if it is validated in larger trials^[4–6]^.

The emergent clinical-development trajectory of YCT-529 has important implications for family planning and public-health frameworks. By targeting spermatogenesis directly rather than modulating systemic gonadal hormones, YCT-529 offers a non-hormonal male contraceptive alternative that could broaden contraceptive choice and promote shared reproductive responsibility^[4]^ (see Table 1). In many settings, contraceptive options remain heavily skewed toward women; introducing a safe, reversible, oral male method may help redistribute responsibility and reduce the disproportionate burden of side-effects, access limitations, and decision-making that currently fall on women.

**Table 1:** Comparison of existing and emerging male contraceptive approaches

Approach	Mechanism of action	Efficacy	Reversibility	Key limitations
Condoms	Physical barrier preventing sperm deposition	~85–98%^[3]^	Immediate	User-dependent failure; compliance issues
Vasectomy	Surgical occlusion of the vas deferens	>99%^[3]^	Limited (requires surgical access)	Invasive; not reliably reversible
Hormonal male contraceptives	Suppress gonadotropins to inhibit spermatogenesis	90–95% (clinical trials)	Gradual recovery	Mood changes, weight gain, reduced libido
YCT-529	Selectively blocks vitamin-A-dependent spermatogenesis pathways	~99% (preclinical animal models)^[2,4]^	Full recovery post-treatment	Long-term safety and human efficacy under evaluation

Condom and vasectomy efficacy, WHO fact sheets and systematic reviews^[3]^; YCT-529 preclinical efficacy, Mannowetz *et al* and Shi *et al* (preclinical/primates)^[2,4]^; YCT-529 early human safety/PK, single ascending-dose study (ClinicalTrials.gov NCT06094283; Communications Medicine) and ongoing repeat-dose studies (ClinicalTrials.gov NCT06542237). ClinicalTrials.gov entries accessed 2 November 2025.

From a population-health perspective, expanding male contraceptive options could contribute to reductions in unintended pregnancies, which remain globally significant in magnitude and cost^[3]^ (see Table 1 for a concise comparison of efficacy and reversibility). Further, progress in this domain aligns directly with the United Nations Sustainable Development Goal (SDG) 3.7, which calls for “ensuring universal access to sexual and reproductive health-care services, including family-planning” by 2030^[7]^. Proactive integration of male contraceptive methods into reproductive-health policy would signal a shift toward more equitable and inclusive family-planning infrastructures.

Nevertheless, ethical, cultural, and behavioral dimensions warrant attention. Male uptake of contraceptive methods may be influenced by sociocultural norms regarding fertility, masculinity, and contraceptive responsibility. Effective public-education campaigns and targeted communication strategies will be essential to ensure informed decision-making, promote partner dialogue, and attenuate potential biases or misconceptions surrounding novel male contraceptives.

Although preclinical and early-phase human data for YCT-529 are highly encouraging, several challenges remain before widespread clinical implementation. First, long-term safety must be convincingly established while animal studies report full reversibility of fertility and minimal off-target toxicity^[2,4]^, while human data remain limited to single ascending-dose studies with no prolonged exposure^[8]^. Repeat-dose human trials of longer duration will be necessary to define the onset of contraceptive efficacy, time to recovery of fertility after discontinuation, and potential rare adverse events (including those involving retinoid pathways in extra-testicular tissues) (see Table 1 for comparative efficacy and reversibility data).

Second, real-world adherence and acceptability may influence effectiveness. The pharmacodynamics of spermatogenesis inhibition imply a latency period before full contraceptive effect and a delay to recovery upon cessation; real-world behaviors (missed doses, irregular usage) must therefore be factored into programmatic planning. Third, health-system integration and policy frameworks must ensure equitable access. Moreover, inclusion of a male oral contraceptive into family-planning programs will require regulatory approval, cost-reimbursement strategies, provider training, and supply-chain logistics particularly to avoid deepening contraceptive inequities in low- and middle-income regions.

In summary, YCT-529 represents a scientifically plausible and socially meaningful advance in male contraception. Provided that upcoming clinical trials validate its safety, efficacy, and reversibility in diverse human populations, this agent has the potential to redefine contraceptive choice and balance reproductive responsibility across sexes. Continued research investment, policy integration, and multidisciplinary dialogue are essential to translate this innovation into equitable clinical practice. This article aligns with the TITAN Guidelines on the need for transparency in AI use in healthcare^[9]^.

## Data Availability

Not applicable.
